# Trends and determinants of HIV/AIDS knowledge among women in Bangladesh

**DOI:** 10.1186/s12889-016-3512-0

**Published:** 2016-08-17

**Authors:** Sanni Yaya, Ghose Bishwajit, Georges Danhoundo, Vaibhav Shah, Michael Ekholuenetale

**Affiliations:** 1 School of International Development and Global Studies, Faculty of Social Sciences, University of Ottawa, Ottawa, ON Canada; 2 School of Medicine and Health Management, Tongji Medical College, Huazhong University of Science and Technology, Wuhan, Hubei China; 3 Faculty of Health, York University, Toronto, ON Canada; 4 Interdisciplinary School of Health Sciences, Faculty of Health Sciences, University of Ottawa, Ottawa, ON K1N 6N5 Canada; 5 The Women’s Health and Action Research Centre, Benin City, Nigeria

**Keywords:** HIV knowledge, Women health, Global health, Bangladesh, Demographic and health survey

## Abstract

**Background:**

Globally, women share an indiscriminate burden of the HIV epidemic and the associated socioeconomic consequences. Previous studies have demonstrated a positive correlation between levels of HIV knowledge with its prevalence. However, for Bangladesh such evidence is non-existent. In this study, we aimed to explore the extent of HIV knowledge in relation to the socio-demographic variables such as age, region, area of residence i.e., urban or rural, wealth index and education, and investigate the factors influencing the level of HIV knowledge among Bangladeshi women.

**Methods:**

We used data from the Bangladesh Demographic and Health Survey (BDHS) survey conducted in 2011. In total 12,512 women ageing between 15 and 49 ever hearing about HIV regardless of HIV status were selected for this study. HIV knowledge level was estimated by analyzing respondents’ answers to a set of 11 basic questions indicative of general awareness and mode of transmission. Descriptive statistics, cross-tabulation and multinominal logistic regression were performed for data analysis.

**Results:**

Little over half the respondents had good knowledge regarding HIV transmission risks. The mean HIV knowledge score was −0.001 (SD 0.914). Average correct response rate about mode of transmission was higher than for general awareness. Educational level of women and sex of household head were found to be significantly associated with HIV knowledge in the high score group. Those with no education, primary education or secondary education were less likely to be in the high score group for HIV knowledge when compared with those with higher than secondary level of education. Similarly those with male as household head were less likely to be in the higher score group for HIV knowledge.

**Conclusions:**

Level of HIV knowledge among Bangladeshi women is quite low, and the limiting factors are rooted in various demographic and household characteristics. Education and sex of the household head have been found to be significantly correlated with the level of HIV knowledge and propound sound grounds for their incorporation in the future HIV prevention strategies. Education of women may also have wider ramifications allowing reduction in gender inequality, which in turn favors higher knowledge about HIV.

## Background

Women in Bangladesh, similar to the current global trend, share a greater risk of HIV infection and mortality compared to men [1]. As per 2012 UNICEF estimates, the prevalence (per 10,000) of HIV among women Bangladeshi was 2.7 against total prevalence of 3.1. Apart from greater risk of susceptibility, the consequence of the disease also has a heavier onus on women owing to their less advantaged socioeconomic standing and lack of physical and social access to quality care especially sexually and reproductive health (SRH). There is a growing consensus on the fact that gender inequality is a great contributor to women’s increased susceptibility to HIV morbidity and its consequences [2–5]. Not surprisingly, the gender aspect of this disease has been able to draw considerable attention in high level international events.

The 2001 United Nations General Assembly Special Session on HIV/AIDS (UNGASS) declaration marked a renewed call for strengthening policy capacities to address the gender issues related to HIV [6]. The problem is complex and exerts a huge toll on not only woman and her children/household, but also retards national progress at large. The negative impacts of gender inequality on HIV prevention efforts are felt even in developed countries [7] despite the greater socioeconomic freedom among women and presence of gender sensitive health and social policies. The major hurdle for such accomplishments in developing countries, like in Bangladesh, is the lack of recognition of the problem and of infrastructural capacity to conduct quality research and thereby identifying the issues that underlie the problem. Development and advancement of gender and health promotion policies are contingent primarily upon availability of workable insights in which Bangladesh lags significantly behind than expected.

The scientific literature on gender aspects of HIV suggests that the root goes deep into the sociocultural value system [8], and is generally attributed to values and perception regarding health and disease especially adult health issues and cultural context of women’s role in the family and society [9, 10]. Level of knowledge of adult and reproductive health, including STDs tend to be higher in societies where people enjoy greater degree in freedom of communication, seek health advocacy, and engage in peer communication [11–13]. Policies that encourage such advantages would contribute to improvement of HIV knowledge among women especially in developing countries where women have little exposure to modern communication tools.

Given the rapid expansion of electronic media and social networking, the social barrier to knowledge improvement regarding STDs is shrinking [14–16], however physical access requires more pertinent policy approaches. In Bangladesh, mobile and internet subscription rate have increased substantially in last two decades. One study encompassing information from 137 countries figured that the gender gap on HIV knowledge has been narrowing; however, knowledge regarding preventive techniques is still low [17]. The same study reported that among people aged 15–24; the rate of comprehensive knowledge about HIV prevention was lower among women compared to men (36 % among women vs 40 % among men) [17].

Despite the well-acknowledged gender aspects of the epidemic, and the possibility of spread of HIV epidemic, more extensive and strategic efforts to promote knowledge and awareness regarding HIV among women in Bangladesh are warranted. Given the relatively low HIV morbidity and mortality rate in Bangladesh, which is similar to other the South Asian countries, there seems to be an apparent oversight of the possibility of its future expansion. Since the first HIV case recognition in the country in 1986, some 9000 people are reported to living with HIV presently. However, it should be noted that though the disease burden is still low, the possibility of spread remains quite high due to widespread prevalence the risk factors [18–20]. The main concerns for HIV transmission in Bangladesh includes high rate of commercial sex, poor blood screening facilities, injection drug use, increasing rate of MSM, all of which deserve serious concerns in their own merit [21–24].

Studies have found that these factors themselves are more prevalent in societies with higher rate of poverty, illiteracy and gender inequality, which are the characteristic of the current situation in Bangladesh [24–27]. Previous studies reported low level of knowledge and awareness regarding STDs among Bangladeshi women [17]. From the perspective of its ease of transmission, level of personal knowledge and awareness are crucial elements for prevention of transmission irrespective of the performance of the healthcare system. Lack of knowledge is usually associated with stigma and leads to poor health seeking behavior, and thus increases the chances of going undiagnosed [28–30]. In Bangladesh, community based knowledge and awareness programs have proven successful in the areas of reducing maternal and tuberculosis mortality. Since women share greater vulnerability to HIV, increasing HIV knowledge among women offers potential opportunities for long-term controlling of the epidemic in Bangladesh. In this study, we aimed to explore the factors associated with the level of HIV knowledge.

Advances in HIV research are attributed to biological, clinical science. However, attention to behavioral risk factors offers the opportunity to explore avenues for intervention development. The future direction of this pandemic depends on the level of knowledge of how the virus is spread and changes in sexual behavior and attitudes. Accordingly, this study aims to explore the impact of socidemographic and household variables on the degree of HIV knowledge among women. Unlike other studies focusing on the clinical aspect of HIV, this paper focuses on the social factors, which could be targeted toward future prevention strategies. The paper being one of the few known population based studies on the subject of HIV knowledge and social factors heralds the research on the topic for future studies as well.

## Methods

### Ethical clearance

The DHS Program maintains strict standards for protecting the privacy of all participants. Before each interview, interviewers read out an informed consent statement and mentioned that participation is voluntary and respondent has the liberty to terminate the interview at any point. In addition, the ICF International ensures that the survey complies with the U.S. Department of Health and Human Services regulations for the protection of human subjects, and the host country ensures that the survey complies with laws and norms of the nation [23]. Approval for this study was not required since the data is secondary and is available in the public domain. More details regarding DHS data and ethical standards are available at: http://goo.gl/ny8T6X.

### Data source, study area, and sampling procedure

Data for this study were sourced from the sixth round (latest) of Bangladesh Demographic and Health survey (BDHS). The DHS survey is nationally representative, cross-sectional in nature, and was carried out in 2011 from July 8 through December 27. The National Institute of Population Research and Training (NIPORT), a renowned health research organization in Bangladesh, conducted the survey. The survey is a part of the International Demographic and Health Survey program known as MEASURE DHS, which is currently active in 90 countries, and conducted under the auspices of the United State Agency for International Development (USAID) and technical assistance of ICF International of Calverton based in USA. BDHS 2011 covered in total 17,141 households.

The survey employed a two-stage cluster sampling method covering the population residing in non-institutional dwelling units in Bangladesh. The country has seven administrative regions, which are subdivided into zilas (districts) and upazilas (townships). At urban level, an upazila is divided into union parishads and mouzas (subdivision of union parishads), and at rural level into wards and mohallas (subdivision of wards). The two-stage clustering of the population involved labelling the smallest administrative units as enumeration areas (EAs) or clusters, each consisting of households at mouza or mohalla level. Firstly, 600 EAs were selected based on their size proportional to that of the units. Of these 600 EAs 393 belonged to the rural areas whereas 207 were from urban areas. A complete listing of housing was done for the selected EAs. In the second stage, households were selected from each EA to ensure effective sampling with an average of 30 households from each EA. A total of 18,000 households were selected for the survey with the expectation of interviewing 18,000 women in the age range of 15–49 years. DHS provides no exact information on the spatial dimension of EAs. However, it consists about hundred to 30 thousand households varying from country to country.

### Variables selection and measurement

The dependent variable in this study was level of HIV Knowledge. A set of 11 questions pertinent to HIV knowledge was selected from DHS questionnaire to which respondents could answer: yes/no/don’t know. Categorical Principal Component analysis was conducted and the variables were quantified and produced object scores for each individual case. With all the component loadings being positive the object scores identified the performance of each individual case in the given domain.

Object scores were calculated using Categorical PCA, which were then ranked into tertiles i.e., Low, Medium and High score on the dimension of HIV knowledge with 3 designating low, 2 medium and 1 as high score.

To facilitate the selection of relevant covariates in the context of HIV knowledge, an extensive literature review was conducted focusing on two most proximate themes: demographic and household status. Based on the availability of variables in the dataset, the following items were selected for analysis: Age, Region, Type of residency i.e., Urban or Rural, Educational Level and Household wealth index.

Respondents’ age in five year groups was used a categorical variable. Type of residency was categorized as rural and urban. Educational status was categorized as having no education, primary education, secondary education and those having higher education. We also included household wealth index in our analysis as it has been shown to be a reliable indicator of household economic condition impacting overall living and health status. DHS surveys assign five different levels based on overall income and wealth status as: poorest, poorer, middle, richer, richest.

### Data analysis

Frequencies and percentages were measured for the questions used in assessing HIV knowledge among participants. Socio-demographic characteristics were tabled using descriptive statistics. Cross tabulation and ***χ***^**2**^ test were performed for associated probabilities and to check for statistical association between groups based on HIV knowledge score (object score). Response variable was divided into tertiles with ties to the mean, as those with high score, medium score or low score. Given the nature of the dependent variable, we used multinomial logistic regression analysis to adjust for potential confounders and examine the independent effect of the explanatory variables on HIV knowledge status. At this level, only the variables, which were found to have significant association from cross-tabulation, were entered into regression model. Odds ratios were measured to assess the likelihood of having high, medium or low score on HIV knowledge among groups with particular socioeconomic and other characteristics. Results of regression analysis are presented as *p*-values and odds ratios. Level of statistical significance was set at *p* < 0.05 (two-tailed). All analyses were performed using SPSS version 21.0 for Windows (SPSS Inc. Chicago. IL. USA).

## Results

### Level of HIV Knowledge

Levels of HIV knowledge are shown in Table 1. Composite level of HIV knowledge i.e., HIV score, was calculated by quantifying the categorical variables measuring HIV knowledge. The mean HIV knowledge score was −0.001 (SD 0.914). HIV knowledge was divided into tertiles with 33.1 % in the high score tertile, 41.9 % in the medium score tertile and 25 % in the low score tertile. The correct response rate about mode of transmission was higher than for general awareness. Percentage of correct answers for individual questions is presented in Table 2. Nearly two-third (70.1 %) of the respondents reported ever hearing about HIV and of those two third 74 % knew that having one sex partner reduces risk of getting HIV. About two-third women knew that condoms could help prevent sexual transmission of HIV. Only about 70 % women knew that HIV could be transmitted during delivery. Over 80 % answered correctly about HIV transmission risks during pregnancy and breastfeeding. About 22 % of the respondents believed that HIV could be caused by means of witchcraft or supernatural powers. Over 90 % knew that using unsterilized needle or syringe and unsafe blood transfusion increases the risk of HIV infection. Lowest correct response was for the questions of whether or not transmission can happen through mosquito bite and sharing food with persons who has HIV (47.3 and 55.6 % respectively).

**Table 1:** Percentage of correct answers by questions among women who reported ever hearing about HIV

Questions	Percentage of correct answers
Reduce risk of getting HIV: always use condoms during sex	64.1
Reduce risk of getting HIV: have one sex partner only, who has no other partners	74.0
A healthy looking person can have HIV	71.3
HIV transmitted during pregnancy	85.4
HIV transmitted during delivery	70.3
HIV transmitted by breastfeeding	81.3
Can get HIV from mosquito bites	47.3
Can get HIV by sharing food with person who has AIDS	55.6
Can get HIV by witchcraft or supernatural means	78.8
Can get HIV by using unsterilized needle or syringe	92.6
Can get HIV through unsafe blood transfusion	91.8

**Table 2:** Socio-demographic characteristics of the participants (*n* = 12512)

Variable	Frequency	Percent
Age		
15–19	1441	11.5 %
20–24	2724	21.8 %
25–29	2562	20.5 %
30–34	1912	15.3 %
35–39	1524	12.2 %
40–44	1292	10.3 %
45–49	1001	8.0 %
Residency		
Urban	5182	41.4 %
Rural	7330	58.6 %
Educational attainment		
Nil	1880	15.0 %
Primary	3418	27.3 %
Secondary	5754	46.0 %
Higher	1460	11.7 %
Region		
Barisal	1521	12.2 %
Chittagong	2028	16.2 %
Dhaka	2335	18.7 %
Khulna	2157	17.2 %
Rajshahi	1727	13.8 %
Rangpur	1445	11.5 %
Sylhet	1299	10.4 %

### Socio-demographic characteristics of the sample population

Socio-demographic characteristics of the sample population are shown in Table 2. Two-third of the participants (65.3 %) were of rural origin and almost a quarter (26 %) had no formal education. About one-third (29.9 %) of the participants had completed primary education and 35.9 % had completed secondary level of education. Only about 8 % (8.2 %) had higher than secondary level of education. About 90 % were from male-headed households. Participants were fairly equitably distributed in the various regions of the country with the top three regions in terms of frequency of participants being from Dhaka followed by Khulna and Chittagong.

Results of cross-tabulation are presented in Table 3 showing the comparison between participants in groups based on their HIV knowledge score in relation to socio-demographic variables (Age, residency, education, region) and household variable (wealth index). Results indicate that household factor i.e., wealth index was significantly associated with level of HIV knowledge. Participants’ age was not a significant factor. Region of residence, type of residence and sex of household head were all significantly associated with level of HIV knowledge. Apart from participants age; owning a mobile phone, literacy status, belonging to mother’s club, and being aware of community clinic were found to be insignificant and were eliminated from subsequent analysis.

**Table 3:** Factors correlated with level of HIV knowledge

	HIV Knowledge Score			Chi square	*p* value
	High Score	Medium Score	Low Score		
Age					
15–19 years	5.2 %	1.9 %	4.4 %	17.549	0.130
20–24 years	10.1 %	3.7 %	8.1 %		
25–29 years	9.7 %	3.6 %	7.3 %		
30–34 years	7.6 %	2.7 %	5.0 %		
35–39 years	6.0 %	2.0 %	4.2 %		
40–44 years	4.9 %	1.8 %	3.6 %		
45–49 years	3.7 %	1.4 %	2.9 %		
Wealth Index					
Poorest	4.5 %	2.0 %	4.1 %	72.757	<0.0001^*^
Poor	6.1 %	2.3 %	6.0 %		
Middle	8.9 %	3.5 %	6.8 %		
Rich	11.8 %	4.0 %	8.7 %		
Richest	15.9 %	5.3 %	10.0 %		
Highest Education					
No Education	5.7 %	2.8 %	6.5 %	159.963	<0.0001^*^
Primary	12.1 %	4.8 %	10.4 %		
Secondary	22.7 %	7.8 %	15.5 %		
Higher	6.8 %	1.6 %	3.3 %		
Region					
Barisal	6.8 %	1.8 %	3.5 %	85.210	<0.0001^*^
Chittagong	7.2 %	2.7 %	6.2 %		
Dhaka	8.4 %	3.5 %	6.8 %		
Khulna	8.0 %	3.1 %	6.1 %		
Rajshahi	6.3 %	2.5 %	5.0 %		
Rangpur	5.9 %	1.7 %	4.0 %		
Sylhet	4.6 %	1.7 %	4.1 %		
Type of Residence					
Urban	20.3 %	6.9 %	14.1 %	13.359	0.001^*^
Rural	26.9 %	10.2 %	21.5 %		
Sex of Household Head					
Male	41.9 %	15.6 %	32.1 %	12.201	0.002^*^
Female	5.4 %	1.5 %	3.6 %		

*Significant at *p* < 0.05

**Table 4:** Factors influencing HIV knowledge among women ageing between 15 and 49 in Bangladesh. 2011

		B	S.E.	Sig.	OR	95 % CI	
						Lower	Upper
High Score	Intercept	0.986	0.256	0.000			
	Age						
	15–19 years	−0.276	0.097	0.004	0.759	0.627	0.917
	20–24 years	−0.197	0.087	0.024	0.821	0.693	0.974
	25–29 years	−0.120	0.086	0.165	0.887	0.749	1.051
	30–34 years	0.041	0.089	0.645	1.042	0.875	1.241
	35–39 years	0.041	0.092	0.654	1.042	0.870	1.248
	40–44 years	0.030	0.095	0.749	1.031	0.856	1.242
	45–49 years	0^b^					
	Residence						
	Urban	0.016	0.047	0.728	1.016	0.927	1.114
	Rural	0^b^					
	Highest education						
	No education	−0.806	0.089	0.000	0.447	0.375	0.531
	Primary education	−0.513	0.077	0.000	0.599	0.514	0.697
	Secondary education	−0.242	0.070	0.001	0.785	0.684	0.901
	Higher education	0^b^					
	Sex of household head						
	Male	−0.150	0.066	0.024	0.861	0.756	0.980
	Female	0^b^					
	Wealth Index						
	Poorest	−0.105	0.084	0.212	0.901	0.764	1.061
	Poor	−0.270	0.075	0.000	0.763	0.659	0.884
	Middle	−0.061	0.068	0.369	0.941	0.824	1.075
	Rich	−0.053	0.059	0.371	0.949	0.845	1.065
	Richest	0^b^					
	Region						
	Barisal	0.539	0.087	0.000	1.713	1.444	2.033
	Chittagong	−0.026	0.079	0.746	0.975	0.834	1.139
	Dhaka	0.083	0.078	0.284	1.087	0.933	1.265
	Khulna	0.166	0.079	0.036	1.180	1.011	1.379
	Rajshahi	0.113	0.083	0.171	1.120	0.952	1.318
	Rangpur	0.297	0.086	0.001	1.346	1.136	1.595
	Sylhet	0^b^					
Moderate Score	Intercept	−1.189	0.364	0.001			
	Age						
	15–19 years	−0.222	0.127	0.081	0.801	0.625	1.028
	20–24 years	−0.144	0.113	0.202	0.866	0.693	1.081
	25–29 years	−0.051	0.112	0.651	0.951	0.764	1.184
	30–34 years	0.042	0.116	0.714	1.043	0.831	1.309
	35–39 years	−0.031	0.120	0.795	0.969	0.765	1.227
	40–44 years	0.036	0.123	0.771	1.037	0.815	1.319
	45–49 years	0^b^					
	Residence						
	Urban	−0.037	0.062	0.549	0.964	0.853	1.088
	Rural	0^b^					
	Highest education						
	No education	−0.106	0.119	0.374	0.900	0.713	1.136
	Primary education	−0.008	0.107	0.943	0.992	0.805	1.223
	Secondary education	0.111	0.098	0.255	1.118	0.923	1.354
	Higher education	0^b^					
	Sex of household head						
	Male	0.157	0.093	0.090	1.170	0.976	1.402
	Female	0^b^					
	Wealth Index						
	Poorest	−0.005	0.107	0.961	0.995	0.806	1.227
	Poor	−0.289	0.099	0.004	0.749	0.616	0.910
	Middle	−0.001	0.088	0.988	0.999	0.841	1.187
	Rich	−0.135	0.078	0.086	0.874	0.750	1.019
	Richest	0^b^					
	Region						
	Barisal	0.275	0.118	0.019	1.317	1.045	1.659
	Chittagong	0.067	0.105	0.526	1.069	0.870	1.314
	Dhaka	0.220	0.101	0.030	1.246	1.021	1.519
	Khulna	0.235	0.104	0.024	1.265	1.032	1.550
	Rajshahi	0.209	0.108	0.054	1.233	0.997	1.525
	Rangpur	0.048	0.118	0.684	1.049	0.833	1.322
	Sylhet	0^b^					

Reference category is High Score. 0^b^ is reference category * Significant at *p* < 0.05

*OR* Odds ratio

### Factors affecting level of HIV knowledge

Factors influencing HIV knowledge among women are shown in Table 4. Based on the result of chi-square test of association, the following variables were found to be significantly associated with the outcome variable and were entered in the regression model: sex of household head, wealth index, education level, region and type of residence. Age of the respondent although not significantly associated, was entered as an independent variable to understand the impact of individual age ranges on HIV knowledge. Women from male-headed families were 0.756 times less likely to have higher score on HIV knowledge as compared to low score. Household wealth was also positively correlated with HIV knowledge level. Women from all the four wealth index quintiles ranging from poorest to richer quintiles were less likely to be in higher score group for HIV knowledge as compared to low score group, compared to richest wealth index quintile. The respective odds ratio for the same were 0.901, 0.763, 0.941 and 0.949, respectively.. Education was strongly associated with HIV knowledge. Those who had education of secondary or less were consistently less likely to be in the higher score group compared to low score group of HIV knowledge when compared with those who had higher than secondary level of education. Previous studies have highlighted the import of education and gender inequity, which resonates, with the findings of the current study [31]. Similarly, region of residence was another variable associated with HIV knowledge. Although, all the regions were not significantly associated with HIV knowledge, Barisal and Khulna consistently were more likely to be in High score or moderate score when compared to Sylhet. Age, although not significantly correlated as an independent variable, women in the age ranges 15–19 and 20–24 were significantly less likely to be the higher score group as compared with the low score group of HIV knowledge, compared to women from 44 to 49 years age range. This provides strategic direction as to the focus of prevention strategies for HIV.

## Discussion

Despite its current low rate, the risk of further expansion of HIV remains high in Bangladesh due to widespread prevalence of the risk factors [17, 22, 23, 32]. Special policy attention will be required to make proper use this opportunity to curb further spread in the country. To facilitate the task, a key step will be conducting large-scale epidemiological studies exploring the direct and remotely associated risk factors especially peoples’ awareness regarding the causes and consequences of the epidemic. Prior studies have demonstrated that prevalence is significantly higher among people who are unaware of the potential routes of transmission [33, 34]. From this perspective, developing a profound understanding on the demographic and sociocultural determinants of peoples’ awareness can set the avenue for evidenced based and effective policy making. To the best of our knowledge, this is the first population-based study on Bangladesh to contribute to the literature from this context. In light of the findings of this study, women’s level of HIV knowledge in Bangladesh is noticeably low. Though most women were aware the major routes of transmission including unsafe blood transfusion and unsterilized needle utilization, there seems to be an overestimation of participants’ perception of risk of transmission through mosquito bites and food sharing with someone who has HIV/AIDS. Respectively 47.3 and 55.6 % women identified these two as risk factors for transmission of HIV. Results imply the necessity for a greater emphasis on clarifying the confusions regarding mode of transmission.

Previous studies have identified education as a ‘social vaccine’ against HIV/AIDS and these findings are corroborated with the findings of the current study were education was found to be significantly correlated with the level of HIV knowledge [31]. In the same vein as that of UNICEF, women’s education has wider repercussions and as per the current study education increases the chances of having higher HIV knowledge.

Contrary to the expectation, household wealth status was not significantly associated with HIV knowledge status. Although the four wealth quartiles, ranging from poorest to richer were less likely to be in the high score group of HIV knowledge than for those who were in the richest quartile, the findings were not statistically significant. The belief that economic solvency ensures access to basic necessities such as proper living condition, education, healthcare services which provides the condition for effective health communication and knowledge acquirement may not be directly playing a role in HIV knowledge, however the indirect impact through education or gender inequity might be of import. Though the country has experienced a substantial rise in per capita income in recent years, 31.5 % of the population lives below national poverty line as per Asian Development Bank, Basic Statistics 2016, especially among women, which reduces women’s capacity to seek for health services. Women from male-headed households having lower HIV knowledge further supports the fact that gender inequity may be playing a role in acquisition of HIV knowledge. In a society fraught with gender discrimination it is assumable that women in male-headed families enjoy less than necessary freedom to engage in adult health education or alike activities that do not align with the concerns of the household head. Such barriers are hard to address by policy making and require change in public attitudes about the value systems in societies. Previous studies have also stressed the importance of gender inequality as factors to be incorporated in ‘positive prevention’ strategies for HIV/AIDS [35].

### Strength and limitations

Our study included a relatively large dataset, was representative of the general population and examined the impact of some very crucial determinants of HIV knowledge. The study was the first of this kind in the context of Bangladesh and invites more detailed and large-scale studies including more culture related determinants. Despite that, our study had few important shortcomings. Utilization of secondary data meant that we had no control over the selection of variables, quality of data, and the measurement of indicators. Moreover, the survey was conducted in 2011; therefore, the current level of HIV awareness may not coincide with our findings.

## Conclusion

This study examined the extent and determinants of HIV knowledge among sexually active women in Bangladesh. The findings indicate that HIV prevention programs have a lot to improve in creating general awareness and clarifying misconceptions regarding mode of transmission of the disease. Education and sex of the household head were significantly correlated with the knowledge of HIV and reaffirms the findings from previous studies as well as indicates the import of their incorporation in the HIV prevention strategies. In the same vein, reducing gender inequality through education can be a viable option for increasing HIV knowledge status. Though being of rural of urban origin showed no significant correlation with HIV knowledge status, rural women are less likely to enjoy the healthcare and counseling facilities compared to their urban counterparts and remain somewhat isolated from the service network. People are more unlikely to seek counseling and treatment for STDs. Social workers have to devise innovative ways to grow trust among people in the services providers and encourage service utilization.

## Abbreviations

BRDB: Bangladesh rural development board
DHS: Demographic and health survey
HIV: Human immunodeficiency virus
STDs: Sexually transmitted diseases
